# Astaxanthin Inhibits H_2_O_2_-Induced Excessive Mitophagy and Apoptosis in SH-SY5Y Cells by Regulation of Akt/mTOR Activation

**DOI:** 10.3390/md22020057

**Published:** 2024-01-24

**Authors:** Tingting Yan, Feng Ding, Yiting Zhang, Yalin Wang, Yinuo Wang, Yuanqingzhi Zhang, Feiyu Zhu, Guanghan Zhang, Xinyi Zheng, Guangyin Jia, Feng Zhou, Yu Zhao, Yan Zhao

**Affiliations:** Department of Bioengineering, Harbin Institute of Technology, Weihai 264209, China; alovelong@aliyun.com (T.Y.); fdhitwh@163.com (F.D.); monica_and@163.com (Y.Z.); aspettandoyl@163.com (Y.W.); wyn020329@163.com (Y.W.); zhangyuanqingzhi@outlook.com (Y.Z.); zfyzoe528@163.com (F.Z.); zhangguanghan853@gmail.com (G.Z.); xyzheng02172003@163.com (X.Z.); guangyinjia2002@gmail.com (G.J.); daflove@126.com (F.Z.); 15944487767@163.com (Y.Z.)

**Keywords:** astaxanthin, H_2_O_2_, Akt/mTOR, apoptosis, mitophagy

## Abstract

Oxidative stress, which damages cellular components and causes mitochondrial dysfunction, occurs in a variety of human diseases, including neurological disorders. The clearance of damaged mitochondria via mitophagy maintains the normal function of mitochondria and facilitates cell survival. Astaxanthin is an antioxidant known to have neuroprotective effects, but the underlying mechanisms remain unclear. This study demonstrated that astaxanthin inhibited H_2_O_2_-induced apoptosis in SH-SY5Y cells by ameliorating mitochondrial damage and enhancing cell survival. H_2_O_2_ treatment significantly reduced the levels of activated Akt and mTOR and induced mitophagy, while pretreatment with astaxanthin prevented H_2_O_2_-induced inhibition of Akt and mTOR and attenuated H_2_O_2_-induced mitophagy. Moreover, the inhibition of Akt attenuated the protective effect of astaxanthin against H_2_O_2_-induced cytotoxicity. Taken together, astaxanthin might inhibit H_2_O_2_-induced apoptosis by protecting mitochondrial function and reducing mitophagy. The results also indicate that the Akt/mTOR signaling pathway was critical for the protection of astaxanthin against H_2_O_2_-induced cytotoxicity. The results from the present study suggest that astaxanthin can reduce neuronal oxidative injury and may have the potential to be used for preventing neurotoxicity associated with neurodegenerative diseases.

## 1. Introduction

Malfunction of the mitochondria occurs with age due to increased oxidative damage, which affects energy utilization and other biological processes in neurons, eventually triggering neuronal death [1]. Mitochondrial failure associated with oxidative stress has been identified as an early pathogenic mechanism contributing to neurodegenerative diseases such as Alzheimer’s disease (AD) and Parkinson’s disease (PD) [2,3]. 

A growing body of studies have indicated that apoptotic cell death plays a critical role in the progression of neurodegenerative diseases [4,5]. The two main apoptotic pathways are the extrinsic pathway, which is initiated by a death ligand binding to a death receptor at the cell membrane, and the intrinsic pathway, which is triggered by the permeabilization of the mitochondrial outer membrane that leads to the release of mitochondrial proteins including cytochrome c and other apoptogenic factors [6]. The mitochondrial outer membrane permeabilization is regulated by B-cell lymphoma-2 (BCL-2) family proteins, among which Bcl-2 is anti-apoptotic, while Bcl-2-associated X protein (Bax) and Bcl-2 antagonist killer (Bak) are pro-apoptotic [7,8]. The elevation of Bax/Bcl-2 and Bak/Bcl-2 ratios correlates with the onset of mitochondria-dependent apoptosis [9]. Both extrinsic and intrinsic apoptotic pathways lead to the activation of caspases that play critical roles in the induction and amplification of apoptotic signals. Caspases can be divided into two categories: initiators (caspase 8 and caspase 9) and executioners (caspase 3, caspase 6, and caspase 7) [10]. The activation of the key effector caspase 3 initiates the apoptotic process and is considered as a marker for apoptosis [11]. 

Mitochondrial quality control mechanisms such as mitophagy are essential for maintaining neuronal health and function [12]. Unusually exuberant mitophagy can promote cell death and is associated with the activation of proapoptotic pathways [7,13]. Excessive mitophagy has been shown to trigger apoptosis via the overexpression of Parkin or enhancing the effect of Parkin via the inhibition of the activation of myeloid cell leukemia-1 (Mcl-1), a unique mitochondrial deubiquitylase [14,15]. Protein kinase B (PKB/Akt)/mammalian target of rapamycin (mTOR) signaling plays vital roles in regulating mitophagy and cell survival. The activation of the phosphoinositide 3-kinase (PI3K)/AKT/mTOR signaling pathway attenuates apoptosis via inhibiting mitophagy [16]. Consistently, the down-regulation of the Akt/mTOR signaling pathway promotes mitophagy-dependent apoptosis that can lead to neuronal loss and cognitive dysfunctions [17,18]. 

Astaxanthin, a carotenoid found in marine organisms, possesses strong antioxidant activity, and has received considerable attention for its beneficial effects in various diseases including cancer, atherosclerosis, cardiovascular disease and neurological diseases [19,20,21,22]. It was shown that astaxanthin reduced H_2_O_2_-induced oxidative damage and apoptosis in mouse macrophage RAW 264.7 [23]. Astaxanthin also inhibited oxidative stress-mediated apoptosis in gastric epithelial cells by reducing reactive oxygen species (ROS) levels and inhibiting the degradation of the DNA repair protein Ku70/80s [24]. In our previous study, astaxanthin protected SH-SY5Y cells against acetaldehyde-induced cytotoxicity by maintaining the redox balance and modulating apoptotic signals [25]. However, the protective mechanisms of astaxanthin against oxidative damage in neuronal cells are still unclear. In this study, human neuroblastoma SH-SY5Y cells were employed to study the protective effects of astaxanthin. It was found that astaxanthin inhibited H_2_O_2_-induced apoptosis by ameliorating mitochondrial damage and reducing mitophagy. It was also shown that the Akt/mTOR signaling pathway was critical for the protective effect of astaxanthin against H_2_O_2_-induced cytotoxicity.

## 2. Results

### 2.1. Effects of Astaxanthin on Cell Survival, Mitochondrial Function and Oxidative Stress in H_2_O_2_-Treated SH-SY5Y Cells

**Figure 1:**
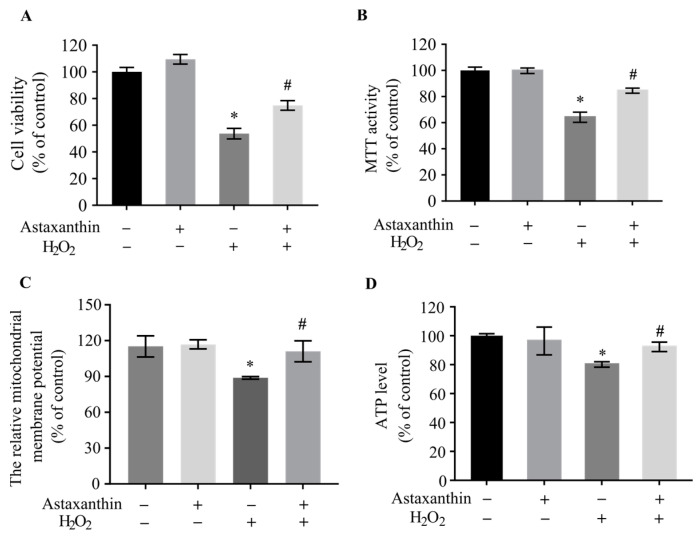
Effects of astaxanthin on cell viability and mitochondrial function in H_2_O_2_-treated SH-SY5Y cells. SH-SY5Y cells incubated with 80 μg/L astaxanthin for 24 h were treated with 500 μmol/L H_2_O_2_ for 2 h. (**A**) Trypan blue, (**B**) MTT, (**C**) mitochondrial membrane potential, and (**D**) ATP examination assays were performed. Data represent the mean ± SD of 6 (**B**) or 3 (**A**,**C**,**D**) independent experiments. * *p* < 0.05 versus control, # *p* < 0.05 versus H_2_O_2_-treated cells.

SH-SY5Y cells were treated with different concentrations of H_2_O_2_, and the 3-(4,5)-dimethylthiahiazo (-z-y1)-3,5-di-phenytetrazoliumromide (MTT) assay was performed. As indicated in Supplementary Figure S1, treatment with H_2_O_2_ at concentrations higher than 500 μmol/L induced a remarkable decline in the MTT activity of SH-SY5Y cells. Astaxanthin treatment alone slightly increased the MTT activity of SH-SY5Y cells at concentrations below 80 μg/L, although the effects were not significant (Figure S2). The trypan blue and MTT assays showed that 500 μmol/L H_2_O_2_ treatment induced cytotoxicity in SH-SY5Y cells, while 80 μg/L astaxanthin significantly protected cells against H_2_O_2_-induced cytotoxicity (Figure 1A,B). Astaxanthin also protected mitochondrial function against oxidative injury induced by H_2_O_2_, leading to the significant recovery of mitochondrial membrane potential (Figure 1C) and preventing a decrease in the adenosine triphosphate (ATP) level (Figure 1D).

**Figure 2:**
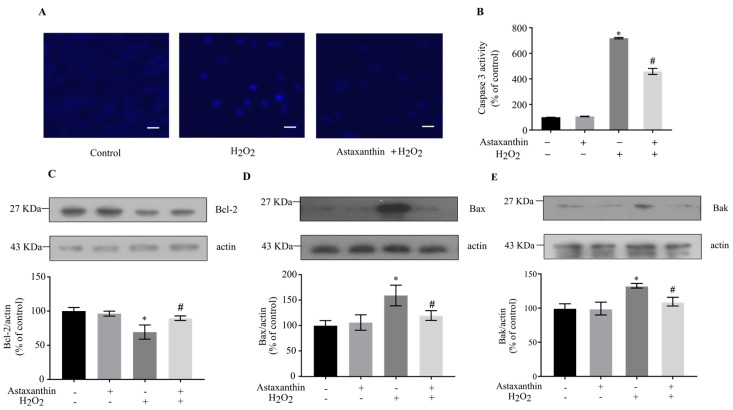
Effect of astaxanthin on apoptosis in H_2_O_2_-treated SH-SY5Y cells. SH-SY5Y cells incubated with 80 μg/L astaxanthin for 24 h were treated with 500 μmol/L H_2_O_2_ for 2 h. (**A**) Cells were stained with Hoechst 33258. Scale bar, 40 μm. (**B**) The caspase 3 activities were determined. (**C**–**E**) Whole-cell lysates were prepared, and the protein levels of Bcl-2, Bax, Bak and actin were determined by means of Western blot analyses. The intensities of the bands were quantified by densitometric analyses and normalized by the amount of actin as indicated in the graphs. Data represent the mean ± SD of 3 independent experiments. * *p* < 0.05 versus control, # *p* < 0.05 versus H_2_O_2_-treated cells.

Neuronal apoptosis is a major pathological process associated with neurological dysfunction in neurons after external stimulation [26]. Hoechst 33258 staining [27] was employed to examine whether H_2_O_2_ induced apoptosis. As indicated in Figure 2A, H_2_O_2_ induced the apoptosis of SH-SY5Y cells, while pre-incubation with astaxanthin significantly decreased the number of apoptotic cells. Caspase 3 is the main executioner of apoptosis, and the activation of caspase 3 is considered as a characteristic of apoptosis [28]. H_2_O_2_ treatment significantly increased the activity of caspase 3, while astaxanthin pre-treatment decreased H_2_O_2_-induced caspase 3 activity by approximately 40% (Figure 2B). As shown in Figure 2C, treatment with H_2_O_2_ decreased the Bcl-2 protein level by nearly 31% in SH-SY5Y cells, while pre-treatment with astaxanthin attenuated the loss of Bcl-2 protein by about 29%. In addition, H_2_O_2_ also significantly increased the levels of Bax and Bak proteins by approximately 60% and 33%, respectively; meanwhile, astaxanthin repressed the upregulation of Bax and Bak by about 25% and 18%, respectively (Figure 2D,E). As shown in Supplementary Figure S3A,B, H_2_O_2_ elevated the ratios of Bax/Bcl-2 and Bak/Bcl-2, which were significantly ameliorated by astaxanthin. These results indicate that astaxanthin might repress apoptosis induced by H_2_O_2_ in SH-SY5Y cells by regulating apoptotic signals. 

**Figure 3:**
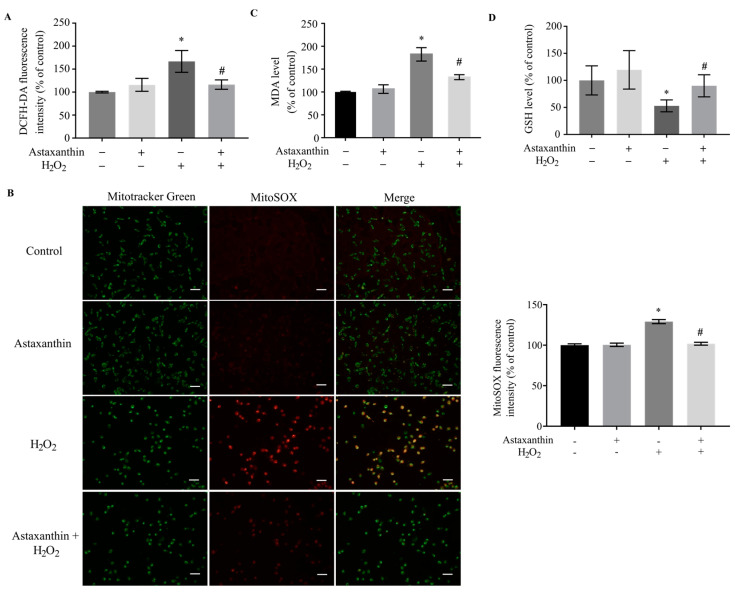
Effects of astaxanthin on oxidative stress in H_2_O_2_-treated SH-SY5Y cells. SH-SY5Y cells incubated with 80 μg/L astaxanthin for 24 h were treated with 500 μmol/L H_2_O_2_ for 2 h. (**A**) ROS, (**B**) mitochondrial ROS, (**C**) MDA, and (**D**) GSH assays were then performed. The MitoSOX fluorescence intensity was analyzed, and the histograms illustrate the values of MitoSOX fluorescence intensity. Scale bar, 40 μm. Data represent the mean ± SD of 3 independent experiments. * *p* < 0.05 versus control, # *p* < 0.05 versus H_2_O_2_-treated cells.

As a potent antioxidant [24], astaxanthin significantly suppressed the ROS production induced by H_2_O_2_ (Figure 3A). Moreover, astaxanthin diminished the H_2_O_2_-stimulated increase in MitoSOX-positive SH-SY5Y cells (Figure 3B), suggesting that astaxanthin reduced the level of ROS in mitochondria. The analyses of MitoSOX fluorescence intensity showed that H_2_O_2_ increased the level of mitochondrial ROS by about 30% compared to the control, while astaxanthin significantly attenuated the increase in mitochondrial ROS by approximately 22%. In addition, the H_2_O_2_-induced production of malondialdehyde (MDA), which was positively correlated with the ROS level, was also decreased by astaxanthin (Figure 3C). Meanwhile, H_2_O_2_ triggered a decline in glutathione (GSH), and astaxanthin ameliorated this effect (Figure 3D). These results suggest that astaxanthin inhibited the oxidative stress damage induced by H_2_O_2_ via ameliorating ROS production and increasing the GSH level.

### 2.2. Effects of Astaxanthin on Mitophagy in H_2_O_2_-Treated SH-SY5Y Cells

**Figure 4:**
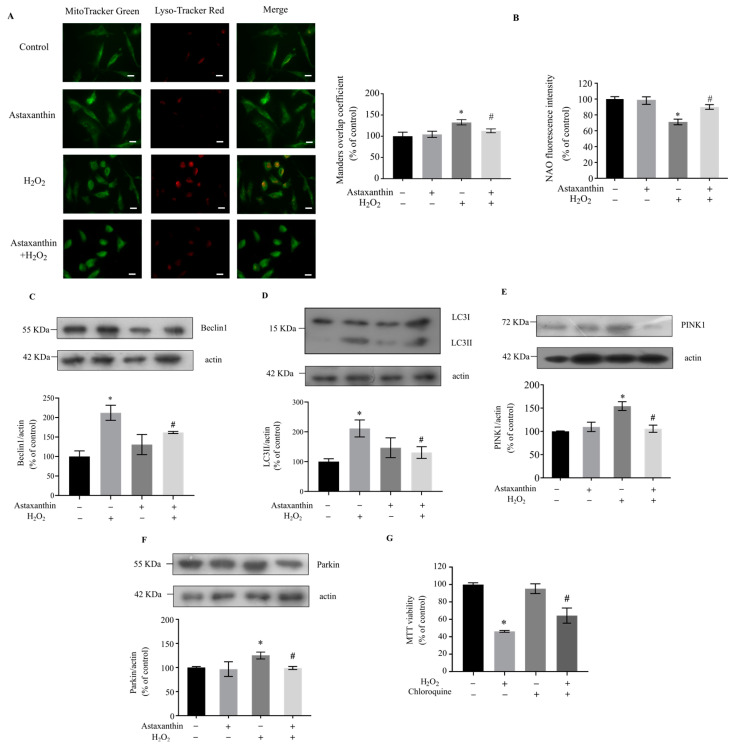
Effects of astaxanthin on H_2_O_2_-induced mitophagy and cytotoxicity in SH-SY5Y cells. SH-SY5Y cells incubated with 80 μg/L astaxanthin for 24 h were treated with 500 μmol/L H_2_O_2_ for 2 h. (**A**) The co-localization of MitoTracker Green and Lyso-Tracker Red was analyzed. The histograms illustrate the values of the Manders overlap coefficient of the fluorescence intensity. Scale bar, 10 μm. (**B**) The NAO staining was performed. (**C**–**F**) Whole-cell lysates were prepared, and the protein levels of Beclin1, LC3, PINK1, Parkin and actin were determined via Western blot analyses. The intensities of the bands were quantified by means of densitometric analyses and normalized by the amount of actin. (**G**) SH-SY5Y cells pre-incubated with 10 μmol/L chloroquine for 24 h were treated with 500 μmol/L H_2_O_2_ for 2 h. An MTT assay was performed. Data represent the mean ± SD of 6 (**G**) or 3 (**A**–**F**) independent experiments. * *p* < 0.05 versus control, # *p* < 0.05 versus H_2_O_2_-treated cells.

ROS produced by damaged mitochondria can trigger mitophagy, which eliminates damaged mitochondria through selective autophagy to maintain the quality of mitochondria [29,30]. The decrease in pH in lysosomes is a prerequisite to complete the mitophagy process [31]. Thus, the co-localization of Mitotracker Green and Lyso-Tracker Red, which specifically labels acidic vesicles (mainly lysosomes) in the cytoplasm, was used to detect mitophagy [32,33]. As shown in Figure 4A, the overlap of the mitochondria and the lysosome was significantly increased by H_2_O_2_ treatment, suggesting that H_2_O_2_ increased mitophagy in SH-SY5Y cells. Astaxanthin treatment significantly reduced the mitophagy induced by H_2_O_2_ (Figure 4A). Next, the mitochondrial mass was measured using the florescent dye 10-N-nonyl acridine orange (NAO), and the results indicated that astaxanthin ameliorated the reduction in mitochondrial mass induced by H_2_O_2_ (Figure 4B). 

Beclin1 is essential for autophagosome formation during mitophagy, and the loss of Beclin1 leads to a reduced rate of mitochondrial clearance [34]. LC3-II, formed by the conjugation of cytosolic LC3-I to phosphatidylethanolamine (PE), participates in the formation of autophagosomes [35]. As shown in Figure 4C,D, the level of Beclin1 was increased by nearly 110%, and the level of LC3II was increased by nearly 120% in cells treated with H_2_O_2_. Astaxanthin decreased the increase in Beclin1 and LC3II in cells treated with H_2_O_2_ by 24% and 38%, respectively. Parkin is a cytosolic E3-ubiquitin ligase, and PINK1 is a mitochondrial serine/threonine-protein kinase [29]. PINK1/Parkin pathway is essential for mitochondrial quality control, which eliminates dysfunctional or damaged mitochondria by recruiting autophagic machinery [36]. As shown in Figure 4E,F, H_2_O_2_ increased the content of PINK1 and Parkin by about 54% and 21%, respectively. Astaxanthin decreased the levels of PINK1 and Parkin in cells treated with H_2_O_2_ by about 30% and 16%, respectively. In addition, chloroquine, an autophagy inhibitor [37], attenuated the cytotoxicity of H_2_O_2_ (Figure 4G), indicating that excessive autophagy might contribute to H_2_O_2_-induced cytotoxicity. Overall, these results show that astaxanthin had a significant inhibitory effect on mitophagy and cytotoxicity induced by H_2_O_2_.

### 2.3. Effects of Astaxanthin on Akt/mTOR Activation in H_2_O_2_-Treated SH-SY5Y Cells

**Figure 5:**
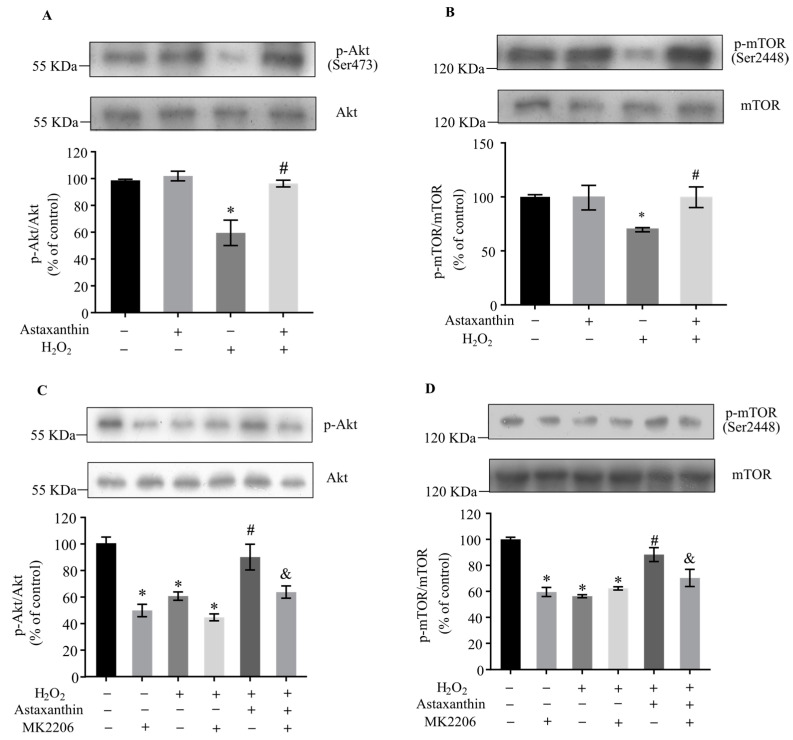
Effects of astaxanthin on Akt/mTOR activation in H_2_O_2_-treated SH-SY5Y cells. (**A**,**B**) SH-SY5Y cells incubated with 80 μg/L astaxanthin for 24 h were treated with 500 μmol/L H_2_O_2_ for 2 h. (**C**,**D**) SH-SY5Y cells incubated with 80 μg/L astaxanthin for 24 h with or without 5 μmol/L MK2206 for 48 h were treated with 500 μmol/L H_2_O_2_ for 2 h. Whole-cell lysates were prepared, and the protein levels of phospho-Akt (Ser473), Akt, phospho-mTOR (Ser2448) and mTOR were determined by Western blot analyses. The intensities of the bands were quantified via densitometric analyses and normalized by the amount of Akt or mTOR, as indicated in the graphs. Data represent the mean ± SD of 3 independent experiments. * *p* < 0.05 versus control, # *p* < 0.05 versus H_2_O_2_-treated cells, & *p* < 0.05 versus cells co-treated with astaxanthin and H_2_O_2_.

**Figure 6:**
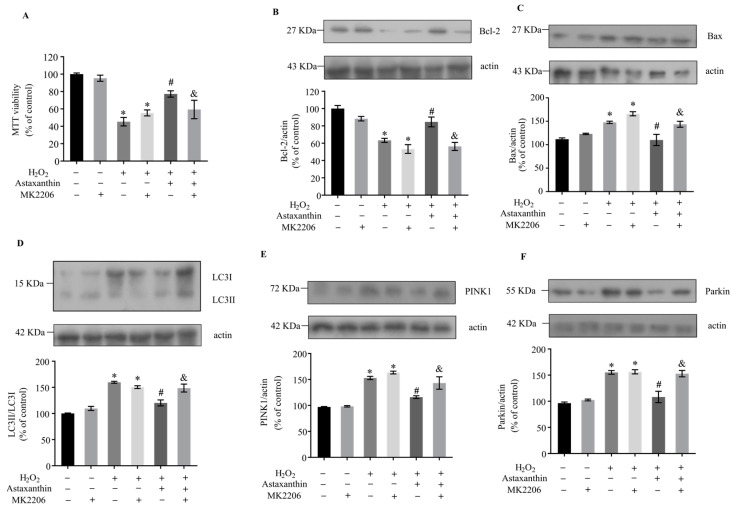
The effects of Akt inhibition on cell survival and the levels of apoptosis and mitophagy-related proteins in SH-SY5Y cells treated with astaxanthin and H_2_O_2_. SH-SY5Y cells incubated with 80 μg/L astaxanthin for 24 h with or without 5 μmol/L MK2206 for 48 h were treated with 500 μmol/L H_2_O_2_ for 2 h. (**A**) The MTT assay was performed. (**B**–**F**) Whole-cell lysates were prepared, and the protein levels of Bcl-2, Bax, LC3, PINK1, Parkin and actin were determined by Western blot analyses. The intensities of the bands were quantified by densitometric analyses and normalized by the amount of actin or LC3I as indicated in the graphs. Data represent the mean ± SD of 6 (**A**) or 3 (**B**–**F**) independent experiments. * *p* < 0.05 versus control, # *p* < 0.05 versus H_2_O_2_-treated cells, & *p* < 0.05 versus cells co-treated with astaxanthin and H_2_O_2_.

The Akt/mTOR signaling pathway is a central regulator of both autophagy and mitophagy [38,39]. In a previous study, astaxanthin pre- and post-treatment increased the protein levels of phospho-Akt and phospho-mTOR in retinal ganglion cells [40]. Here, the activation of Akt and mTOR was determined to examine whether astaxanthin inhibits mitophagy by promoting the Akt/mTOR signaling pathway in SH-SY5Y cells. As shown in Figure 5A,B, H_2_O_2_ decreased the ratios of phospho-Akt/Akt and phospho-mTOR/mTOR by about 40% and 30%, respectively; meanwhile, astaxanthin significantly increased the levels of activated Akt and mTOR by approximately 60% and 48% compared with the H_2_O_2_-treated cells. The inhibitor of Akt, MK2206 [41,42], markedly inhibited the phosphorylation of Akt and mTOR (Figure 5C,D). Meanwhile, MK2206 attenuated the protective effect of astaxanthin against H_2_O_2_-induced cytotoxicity (Figure 6A). Furthermore, MK2206 prevented the modulation of astaxanthin on apoptosis (Figure 6B,C) and mitophagy-related proteins (Figure 6D–F) induced by H_2_O_2_. These results indicate that astaxanthin ameliorated H_2_O_2_-induced mitophagy and apoptosis via activating the Akt/mTOR signaling pathway in SH-SY5Y cells.

## 3. Discussion

Many central nervous system diseases, such as AD, PD and amyotrophic lateral sclerosis (ALS), are associated with a certain extent of redox imbalances and neuronal loss [43,44]. Oxidative stress is associated with DNA damage, mitochondrial dysfunction, chronic inflammation and apoptosis in brain tissue, and it has been considered as a major factor that contributes to the pathogenesis of neurodegenerative diseases [45,46,47]. The mitochondria are the indispensable source of energy production for neurons. The proper function of the mitochondria is essential for physiological functioning and cellular responses to diverse stressors. The mitochondria are the primary source of ROS, and the accumulation of damaged mitochondria plays a critical role in intrinsic apoptosis [48]. SH-SY5Y cells are widely used in the studies of oxidative stress injury and pathological mechanisms of neurodegenerative diseases [49,50,51]. The present study indicated that H_2_O_2_ induced massive ROS production and mitochondrial ROS release in SH-SY5Y cells, decreased mitochondrial membrane potential, and damaged mitochondrial function. In addition, H_2_O_2_ triggered nucleus pycnosis and the activation of caspase 3, raised the ratios of Bax/Bcl-2 and Bak/Bcl-2, and led to the mitochondria-dependent apoptosis pathway. The PINK1/Parkin pathway promotes the selective degradation of damaged mitochondria via mitophagy [7]. Emerging evidence has supported that mitophagy plays a double-edged role in responses to exogenous stressors, contributing to both the protection of cellular homeostasis and the induction of cell death [52,53]. Oxidative stress has been found to induce mitophagy and apoptosis [54]. It has been shown that mitophagy is involved in apoptotic stress responses and promotes apoptosis [53]. Apoptosis can be triggered in various human cells by the overexpression of Parkin or the enhancement of the ubiquitylation effect of Parkin, which can cause the excessive removal of mitochondria [14,15]. At the same time, the suppression of mitophagy by blocking the PINK1/Parkin signaling pathway reduces apoptosis [55]. It was demonstrated in this study that H_2_O_2_ induced excessive mitophagy via the PINK1/Parkin-dependent pathway, while inhibiting mitophagy with chloroquine protected SH-SY5Y cells against the cytotoxicity induced by H_2_O_2_.

Astaxanthin is a potent antioxidant [56,57,58] with neuroprotective effects, but the underlying mechanisms are still unclear. Astaxanthin attenuated subarachnoid hemorrhage (SAH)-induced early brain injury by inhibiting mitochondria-associated neuron apoptosis, improving mitochondrial function and neuronal survival [59]. Astaxanthin was also shown to protect against heat-induced injury in the mouse hypothalamus by reducing mitophagy and apoptosis [60]. Consistently, in the present study, astaxanthin significantly reduced apoptosis while inhibiting mitophagy induced by H_2_O_2_, suggesting that astaxanthin might inhibit apoptosis and cytotoxicity through ameliorating mitophagy.

The class I PI3K/Akt/mTOR signaling pathway is involved in the regulation of both apoptosis and autophagy, which can be induced simultaneously or sequentially [61]. It has been shown that endothelial monocyte-activating polypeptide II induces mitophagy and cytotoxicity in human glioblastoma cells and glioblastoma stem cells through inhibiting the PI3K/Akt/mTOR signal pathway [62]. In addition, the inhibition of Akt/mTOR signaling following mitochondrial stress promotes apoptosis [63]. H_2_O_2_ treatment has been demonstrated to inhibit PI3K/Akt/mTOR activation [64] and induce autophagy and apoptosis [65]. Similarly, this study showed that H_2_O_2_ suppressed the activation of Akt and mTOR and induced mitophagy and apoptosis in SH-SY5Y cells. It was also found that astaxanthin ameliorated the inhibition of the Akt/mTOR signaling pathway induced by H_2_O_2_ and attenuated mitophagy and apoptosis, while pretreatment with MK2206, the inhibitor of Akt, attenuated the protective effect of astaxanthin. Thus, promoting the Akt/mTOR signaling pathway might be an important mechanism contributing to the inhibitory effects of astaxanthin on mitophagy and apoptosis. 

**Figure 7:**
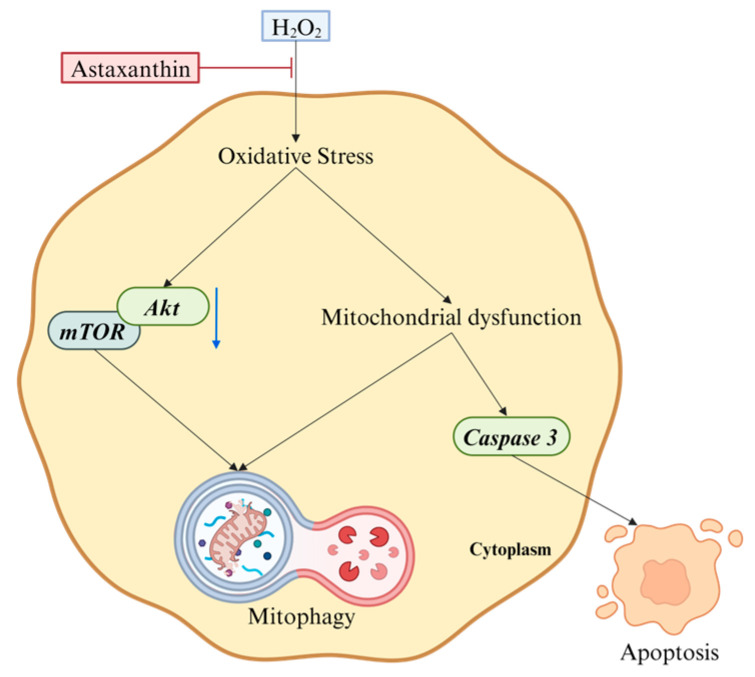
Astaxanthin inhibits H_2_O_2_-induced apoptosis and mitophagy via promoting Akt/mTOR signaling.

In conclusion, as illustrated in Figure 7, the present study demonstrated that astaxanthin inhibited H_2_O_2_-induced apoptosis and cytotoxicity by ameliorating oxidative stress and attenuating excessive mitophagy. Furthermore, astaxanthin might repress mitophagy and apoptosis by promoting of Akt/mTOR signaling.

## 4. Materials and Methods

### 4.1. Materials

Fetal bovine serum (FBS), penicillin, streptomycin, Dulbecco’s modified Eagle’s medium (DMEM), trypsin, MitoTracker™ Green FM, LysoTracker™ Red, NAO staining and MitoSOX™ Red mitochondrial superoxide indicator were purchased from Thermo Fisher Scientific (Rockford, IL, USA). Astaxanthin, MTT, chloroquine and 2′,7′-dichlorodihydrofluorescein diacetate (DCFH-DA) were purchased from Sigma Chemical (St. Louis, MO, USA). MK2206, a BCA protein assay kit, a mitochondrial membrane potential assay kit, a caspase 3 activity kit, an ATP detection assay kit, a Beyo ECL moon Western blotting detection system, Hoechst 33258 dye, horseradish peroxidase (HRP)-labeled donkey anti-goat, goat anti-mouse and goat anti-rabbit IgG (H + L) were purchased from Beyotime Institute of Biotechnology (Shanghai, China). MDA and GSH detection assay kits were purchased from Nanjing Jiancheng Bioengineering Institute (Nanjing, Jiangsu, China). Antibodies for phosphorylated Akt (Ser473) (#9271, 1:1000), Akt (#9272, 1:1000), phosphorylated mTOR (Thr2448) (#2971, 1:1000), mTOR (#2972, 1:1000), LC3 (#12741, 1:1000) and Beclin1 (#3738, 1:1000) were purchased from Cell Signaling Technology (Danvers, MA, USA). Antibodies for PINK1 (sc-518052, 1:1000), Parkin (sc-32282, 1:1000) and actin (sc-8432, 1:1000) were purchased from Santa Cruz Biotechnology (Dallas, TX, USA). Antibodies for Bcl-2 (26593-1-AP, 1:2000), Bax (50599-2-Ig, 1:2000) and Bak (29552-1-AP, 1:2000) were purchased from Proteintech (Wuhan, Hubei, China).

### 4.2. Cell Culture

Human neuroblastoma SH-SY5Y cells, obtained from Shanghai Institutes for Biological Sciences of Chinese Academy of Sciences (Shanghai, China), were cultured in DMEM supplemented with 10% FBS, 1% penicillin and streptomycin. The cells were maintained at 37 °C in a humidified atmosphere with 5% CO_2_ and 95% air. 

SH-SY5Y cells pre-treated with 80 μg/L astaxanthin for 24 h were treated with 500 μmol/L H_2_O_2_ for 2 h. According to our previous study, SH-SY5Y cells were pre-treated with 10 μmol/L chloroquine for 24 h to ameliorate the mitophagy induced by H_2_O_2_ [37]. Treatment with 5 μmol/L MK2206 for 48 h was employed in the present study to attenuate the activation of Akt, according to the procedures described in previous studies with minor modifications [41,42].

### 4.3. Trypan Blue Assay

Cells were seeded at 1 × 10^5^ cells/well in a 6-well plate and cultured in a CO_2_ incubator for 24 h before drug treatment. After treatment, the medium was removed, and the cells were washed twice with phosphate-buffered saline (PBS) and harvested by trypsinization. Cells were then incubated with 0.04% trypan blue solution for 3 min, and the number of live and dead cells was counted with a hemocytometer according to a previous study [66]. The cell viability was calculated using Formula (1).

(1) $$Cell\;Viability\left(\%\right)=\frac{Whole\;Cell\;Nubmer-Dead\;Cell\;Number}{Whole\;Cell\;Nubmer}\times 100\%$$

### 4.4. MTT Assay

For cell viability measurements, cells (4 × 10^3^/well) were seeded in a 96-well culture plate, and the MTT assay was performed according to a previous study with minor modifications [67]. After the treatment, cells were incubated with MTT at 37 °C for 4 h. The formazan formed was then dissolved in dimethyl sulfoxide (DMSO), and the absorbance was measured at 570 nm using a microplate reader.

### 4.5. ATP Assay

The ATP content was measured using a luciferase luminescent ATP detection assay kit according to the manufacturer’s instructions and a previous study with minor modifications [68]. After the treatment, cells were lysed and centrifuged at 12,000× *g* for 10 min at 4 °C. The supernatants or ATP standard solutions were mixed with luciferin and luciferase. Luciferase converted luciferin into oxyluciferin and light, which was proportional to the concentration of ATP present in the reaction mixture. The luminescence was measured using a Synergy HTX Multi-mode Microplate Reader.

### 4.6. Determination of Mitochondrial Membrane Potential

The mitochondrial membrane potential was measured as described previously with minor modifications [69]. After the treatment, cells were incubated at 37 °C for 20 min with the JC-1 working buffer. Cells were washed twice and then harvested by trypsinization. The fluorescence intensity of the cells was measured using a fluorospectrophotometer (excitation wavelength at 490 nm; emission wavelengths at 590 and 525 nm). The relative mitochondrial membrane potential was estimated using the red/green fluorescence intensity ratio.

### 4.7. Measurement of ROS

The levels of ROS were determined using the fluorescent probe DCFH-DA according to the method presented in a previous study with minor modifications [70]. Cells (1 × 10^5^/well) were seeded in 6-well culture plates. After treatment, cells were harvested by trypsinization and incubated with 10 μmol/L DCFH-DA for 30 min at 37 °C in the dark. The fluorescence was then examined using an Olympus BX53 fluorescence microscope.

The detection of mitochondrial ROS was performed as described previously with minor modifications [71]. Cells were co-stained with MitoSOX Red and MitoTracker Green. After treatment, cells were stained with MitoSOX Red mitochondrial superoxide indicator (5 μmol/L) diluted in DMEM with 10% FBS at 37 °C for 15 min. Cells were then stained with MitoTracker Green (180 nmol/L) diluted in FBS-free DMEM at 37 °C for 30 min. The fluorescence signal was observed using an Olympus BX53 fluorescence microscope (10 × objective). The excitation and emission wavelengths of MitoTracker Green and MitoSOX Red are 490 nm/516 nm and 510 nm/590 nm, respectively. ImageJ 2.1.4.7 was employed to merge the images and calculate the MitoSOX fluorescence intensity.

### 4.8. Measurement of MDA

The contents of MDA were measured via the thiobarbituric acid (TBA) assay as described previously with minor modifications [72]. Cells (1 × 10^5^/well) were seeded in 6-well culture plates. Cells were harvested by means of trypsinization, and the cell lysates were prepared using an ultrasonic cell crusher. The levels of MDA in the samples were determined using commercially available kits according to the manufacturer’s instructions. Lipid peroxidation was evaluated by means of the reaction of MDA with thiobarbituric acid to form a product measured at 532 nm using a spectrometer. Protein concentrations were determined via the BCA protein assay, and the levels of MDA were normalized by the amount of protein in the total cell lysates.

### 4.9. Measurement of GSH

Cells were harvested after the treatments by means of trypsinization to determine the concentrations of GSH, and the total cell lysates were collected. The levels of GSH in the samples were determined using the GSH detection assay kit according to the manufacturer’s instructions and the previous study with minor modifications [73]. For assays of GSH, samples were first deproteinized. The levels of GSH were then measured by the reaction of the sulfhydryl group of GSH with 5,5′-dithiobis-2-nitrobenzoic acid (DTNB) to form a yellow-colored product that was measured at 405 nm using a spectrometer. Protein concentrations were determined via the BCA protein assay, and the levels of GSH were normalized by the amount of protein in the total cell lysates.

### 4.10. The NAO Staining

NAO staining was used to detect mitochondria mass according to the previous study with minor modifications [74]. Cells (1 × 10^5^/well) were seeded in 6-well culture plates. After treatments, cells were harvested by means of trypsinization after being incubated with NAO (50 nmol/L) for 30 min at 37 °C in the dark. The fluorescence intensity was analyzed using a Multi-mode Microplate Reader (excitation at 488 nm, emission at 530 nm).

### 4.11. Hoechst 33258 Nuclear Staining

Apoptotic cells were detected by means of the Hoechst 33258 nuclear staining assay according to the previous study with minor modifications [75]. Cells were fixed for 10 min at room temperature after treatments. The fixed cells were incubated with Hoechst 33258 for 5 min at room temperature and subsequently washed with PBS three times in the dark. The fluorescence was examined using an Olympus BX53 fluorescence microscope (excitation at 350 nm, emission at 460 nm) (20 × objective).

### 4.12. Measurement of Caspase 3 Activity

The activity of caspase 3 was determined using a caspase 3 activity kit according to the manufacturer’s instructions and a previous study with minor modifications [76]. Cells were harvested by digesting with trypsin, and the cell lysates were incubated with Ac-DEVD-pNA and the reaction buffer in the kit for 10 h at 37 °C. Substrate cleavage was then measured using a spectrometer at 405 nm.

### 4.13. Western Blot Analyses

The Western blot analyses were performed according to our previous study [37]. The whole-cell lysates were prepared in cell lysis buffer (Tris 20 mmol/L, NaCl 150 mmol/L, EDTA 1 mmol/L, sodium pyrophosphate 2.5 mmol/L, NaF 20 mmol/L, β-glycerophosphoric acid 1 mmol/L, and Na_3_VO_4_ 1 mmol/L) after treatment. Protein concentration was measured using a BCA protein assay kit. The protein extracts (10 μg) were resolved by SDS-polyacrylamide gel electrophoresis and then transferred to polyvinylidenedifluoride (PVDF) membrane. After blocking, the PVDF membrane was incubated with a primary antibody, followed by an HRP-coupled secondary antibody. An enhanced chemiluminescence substrate reaction (Beyo ECL moon Western blotting detection system) was used to detect the protein bands. The intensities of the bands were then quantified by densitometric analyses.

### 4.14. Mitochondria and Lysosomes Colocalization

The fluorescence signals of MitoTracker Green and Lyso-Tracker Red were used to indicate the level of mitophagy as described previously with minor modifications [77]. Cells (2 × 10^5^) were cultured in a 6 cm plate. After treatment, cells were incubated with Lyso-Tracker Red for 15 min at 37 °C and fixed with 4% paraformaldehyde for 15 min. Cells were then incubated with MitoTracker Green for 30 min at 37 °C. The fluorescence signal was observed using an Olympus BX53 fluorescence microscope (100 × objective), and a total of 20 images were randomly taken from each treatment group and then analyzed using ImageProPlus6.0 software. The excitation and emission wavelengths of MitoTracker Green and Lyso-Tracker Red are 490 nm/516 nm and 577 nm/590 nm, respectively. To characterize the co-localization of MitoTracker Green and Lyso-Tracker Red, a total of 30–35 cells were randomly chosen from each treatment group, and the Manders overlap coefficient was calculated. Manders overlap coefficient was calculated using the following Formula (2), where S1*_i_* and S2*_i_* represent the signal intensity of individual pixels in channel 1 (red) and 2 (green), respectively (*i* represents single pixel) [78]. The values from three parallel treatments were then averaged.

(2) $$\frac{\sum_{i}S1_{i}\times S2_{i}}{\sqrt{\sum_{i}{\left(S1_{i}\right)}^{2}\times \sum_{i}{\left(S2_{i}\right)}^{2}}}$$

### 4.15. Statistical Analysis

Quantitative data are analyzed using GraphPad Prism 7.00. Statistical analyses of the data were performed by means of one-way or two-way ANOVA. *p* < 0.05 was considered statistically significant.

## Data Availability

The data presented in this study are available on request from the corresponding author.

## Supplementary Materials

The following supporting information can be downloaded at: https://www.mdpi.com/article/10.3390/md22020057/s1. Figure S1: Effects of different concentrations of H_2_O_2_ on cell viability in SH-SY5Y cells. Figure S2: Effects of different concentrations of astaxanthin on cell viability in SH-SY5Y cells. Figure S3: Effect of astaxanthin on the ratios of Bax/Bcl-2 and Bak/Bcl-2 in H_2_O_2_-treated SH-SY5Y cells.
