# Supplementary material for: Astaxanthin Inhibits H2O2-Induced Excessive Mitophagy and Apoptosis in SH-SY5Y Cells by Regulation of Akt/mTOR Activation
Source: Mar Drugs. 2024 Jan 24;22(2):57. doi: 10.3390/md22020057 (PMC10890442; doi:10.3390/md22020057)
Supplement: Supplementary file 1 [file marinedrugs-22-00057-s001.zip › marinedrugs-2798737-supplementary.pdf]

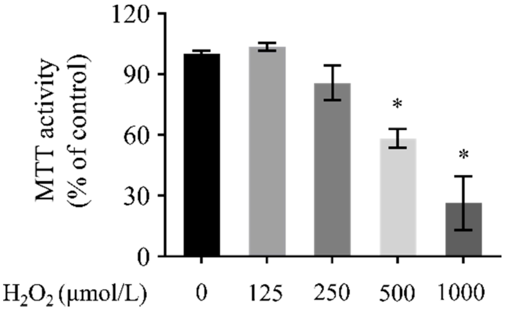

Figure S1. Effects of different concentrations of H_2O_2 on cell viability in SH-SY5Y cells. SH-SY5Y cells were treated with 0, 125, 250, 500 and 1000 μmol/L H_2O_2 for 2 h. MTT assay was then performed. Data represent the mean ± SD of 6 independent experiments. *p<0.05 versus control.

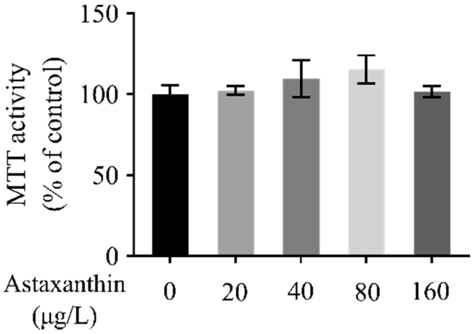

Figure S2. Effects of different concentrations of astaxanthin on cell viability in SH-SY5Y cells. SH-SY5Y cells were treated with 0, 20, 40, 80 and 160 μg/L astaxanthin for 24 h. MTT assay was then performed. Data represent the mean ± SD of 6 independent experiments.

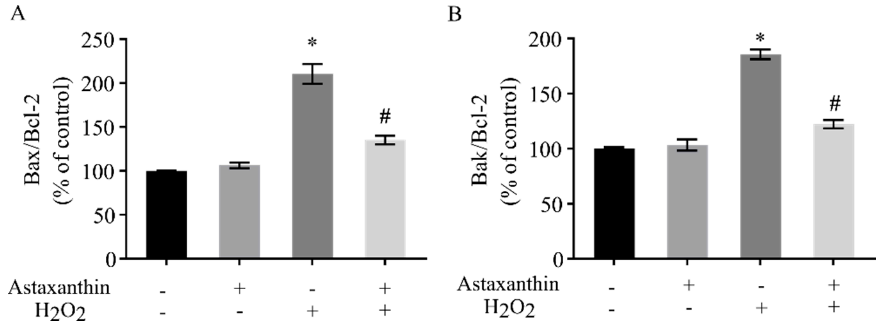

Figure S3. Effect of astaxanthin on the ratios of Bax/Bcl-2 and Bak/Bcl-2 in H_2O_2-treated SH-SY5Y cells. The ratios of Bax/Bcl-2 and Bak/Bcl-2 were calculated using the intensities of Bax, Bak and Bcl-2 adjusted by the intensities of actin, respectively. *p<0.05 versus control, #p<0.05 versus H_2O_2-treated cells.
